# Scapula fractures in complex shoulder injuries and floating shoulders: a classification based on displacement and instability

**DOI:** 10.1186/1752-2897-8-16

**Published:** 2014-11-07

**Authors:** Jan Friederichs, Mario Morgenstern, Volker Bühren

**Affiliations:** Trauma Center Murnau, Prof.-Küntscher-Str. 8, 82418 Murnau, Germany

**Keywords:** Scapula fracture, Floating shoulder, Complex shoulder girdle injury

## Abstract

**Background:**

Scapula fractures with injuries of the Superior Shoulder Suspensory Complex are often referred to as floating shoulders. However, present studies do not allow comparative evidence on indication for surgical treatment mostly due to the lack of precise definitions and comparable classifications. The aim of this study was to retrospectively analyze common types of complex shoulder injuries and develop a feasible classification allowing a therapeutic algorithm.

**Methods:**

The study group consisted of 107 patients with scapula fractures combined with ipsilateral injuries of the shoulder girdle treated in a single trauma center between 2003 and 2010. Three-dimensional computed tomography was analyzed for dislocation and instability and assigned to subgroups of a defined classification system. Clinical data was acquired from a previously established database of all patients treated for the diagnosis of a scapula fracture.

**Results:**

Fifty-seven of 107 (53.3%) complex scapula fractures were non-displaced and stable representing Type A fractures. Depending on the fracture pattern, three subgroups were defined. Treatment of Type A injuries should be non-operative. Displaced fractures of the scapula with a stable shoulder girdle were considered Type B injuries and represented 18.7% of all fractures. Thirty fractures (28%) with an unstable shoulder girdle were classified as Type C injuries. Again, subgroups with common injury patterns were identified. For both groups, operative treatment is recommended.

**Conclusions:**

The described classification system is a proposal able to categorize complex shoulder injuries and allows a comparison of injury patterns in further studies.

## Background

The combination of a fracture of the scapular neck with an ipsilateral fracture of the clavicle is often referred to as a “floating shoulder”. Since its first description [1], many clinical and biomechanical studies have focused on this complex shoulder injury and the definition of a floating shoulder has been updated over the last decades. However, the recent studies do not allow comparative evidence on indication for surgical treatment mostly due to the lack of precise definitions and comparable classifications [2, 3]. The aim of this study was to retrospectively analyze common types of complex shoulder injuries and develop a feasible classification allowing a therapeutic algorithm.

The mechanism of scapular fractures is always a high-energy trauma. Concomitant injuries occur in up to 90% of the patients with the majority being thoracic injuries followed by injuries of the ipsilateral extremity [3–5]. Thus, complex shoulder injuries often involve fractures of the ipsilateral clavicle, the acromion or the coracoid process as well as ligamentous and osseoligamentous structures as the acromioclavicular joint, the coracoclavicular ligaments and the coracoacromial ligament. However, the classification of scapula fractures described by Euler and Ruedi [6] as well as the Ideberg classification of glenoid fractures [7] do not systematically include concomitant injuries of the shoulder girdle. Goss and co-workers introduced the concept of the Superior Shoulder Suspensory Complex (SSSC) and expanded the definition of a floating shoulder to a double disruption of this bone and soft tissue ring [8, 9]. In contrast to previous definitions of a floating shoulder being a combined fracture of the scapular neck and the ipsilateral clavicle [1, 9–11], only a double-disruption of the SSSC causes an unstable anatomical situation and therefore a true floating shoulder [10]. Biomechanical cadaver studies performed by Williams and colleagues emphasized that a fracture of the scapular neck and the ipsilateral clavicle can only produce an instable, floating shoulder when combined with a disruption of the coracoacromial and acromioclavicular capsular ligaments [12]. However, this assertion of stability has recently been doubted [3] indicating that there are still controversial criteria of stability and little agreement on classifications and indications.

Although the management of complex shoulder injuries has changed towards operative fixation strategies [13, 14], there are reports of equal or superior results of a non-operative treatment [15, 16]. Several series propagated early functional treatment after open reduction and internal fixation with excellent results [17, 18], other studies compared groups of conservatively treated patients with surgically treated patients and reported of good outcome in both groups [11, 19, 20].

Nonetheless, evidence-based conclusions and guidelines for surgical indications can not be implied mostly because these complex injuries are not classified to a comparable extent. The decisions whether to operate are based not on clear algorithms but on expert opinion although recently precise criteria for operative treatment have been published mostly based on accepted definitions of displacement [3, 21]. In contrast, there are no clear criteria published for stable or unstable complex shoulder injuries with a double lesion of the SSSC. In addition, there is still disagreement, whether a solitary fixation of the clavicle is able to stabilize the unstable shoulder girdle [9, 11, 18, 22] or if a combined open reduction and internal fixation of both, clavicle and scapula, is necessary.

Although typical injury patterns of scapular fractures have been described recently [3, 23] and the majority of scapular fractures does not involve the scapular neck and thus does not represent the “typical” floating shoulder, there is no known systematic classification of periscapular complex shoulder injuries. The commonly used classifications of scapula and glenoid fractures do not include the concomitant injuries of the ipsilateral shoulder girdle. In our study, we therefore included not only fractures of the scapular neck but all scapular fractures combined with injuries of the ipsilateral SSSC to analyze and classify typical injury patterns.

## Methods

This retrospective study was conducted on the basis of an established database, where all patients with fractures of the scapula treated in our institution are included. All patients treated from January 2003 until December 2010 were included in this study. The study was conducted in compliance with the Helsinki Declaration and according to the guidelines and the approval of the Ethics Committee of the Bavarian Medical Board. Our study group was defined as patients with a complex scapular injury with concomitant injury of the Superior Shoulder Suspensory Complex (SSSC) including trauma of the ipsilateral clavicle, the acromioclavicular joint, the acromial process and the coracoid process. Thus, from a total of 442 patients with the diagnosis of a scapula fracture, a subgroup of 107 patients (24.2%) fulfilled the study criteria of a complex injury named above. All patients included in this study had three-dimensional computed tomography scans performed at least preoperatively. All CT-scans including three-dimensional reconstructions of the scapula were reviewed independently by two surgeons of the investigating team (J.F. and M.M.). Concomitant injuries of the SSSC were documented and the injuries were classified according to the following criteria. Group A consisted of non-displaced fractures with no radiographic signs of instability. Fractures with a significant displacement either of the glenoid or the scapular body and neck and lacking criteria of instability were assigned to Group B. Displacement was defined on expert opinion similar to the criteria of displacement previously described in literature [3, 23] with >2 mm for intra-articular fractures, 10 mm for the superior border and the scapular neck and >20 mm for fractures of the scapula body. Injuries with an instability, thus representing a true floating shoulder, were classified as Type C injuries. Fracture types of the scapula were analyzed and a sub-classification was created based on a systematic description of common fracture patterns. Injuries of the SSSC were reviewed and documented for each patient based on all radiographs, CT-scans, MRI-scans and additional information as intraoperative findings. MRI-scans were performed, if a ligamentous instability was suspected in order to aid the classification and decision. However, this was not routinely performed in all patients.

Clinical data were available for the complete study group. A summary of clinical data is shown in Table 1. In addition, concomitant injuries including vascular injuries and neurologic deficits were monitored. Conservative treatment consisted of a short period (one week) with shoulder immobilizer or sling followed by early physical therapy with radiographic controls after one, two and six weeks. For operative therapy, open reduction and internal plate fixation of the clavicle, the scapula or both was performed and documented for study reasons. Descriptive statistics including percentages, means and standard deviations were calculated for all classified subgroups.

**Table 1:** **Injury pattern and treatment of 107 patients with scapular fractures combined with injuries of the ipsilateral Superior Shoulder Suspensory Complex (SSSC)**

Fracture/Injury	
Scapula	107/107 (100%)
Clavicula	102/107 (95%)
Acromion	22/107 (21%)
Coracoid	30/107 (28%)
AC-joint*	11/107 (10%)
Humerus	9/107 (8%)
Mortality	4/107 (3.7%)
Spinal cord lesion	9/107 (8%)
Brachial plexus lesion	15/107 (14%)
Vascular injury	1/107 (1%)
**Treatment**	
Non-operative	33/107 (31%)
Clavicula	39/107 (36%)
Scapula	20/107 (19%)
Scapula and Clavicula	11/107 (10%)
Others	4/107 (4%)

*Acromioclavicular joint.

## Results

One hundred and seven patients met the criteria of a complex scapula injury with either a fracture of the clavicle, the acromion, the coracoid or a disrupted acromioclavicular joint. The study group consisted of 91% male and only 9% female patients with a median age of 45 years (range 19–76 years, average 46 years). The right side was predominant with 55% of the injuries, the left side was involved in 45% of the patients. As expected, 84% of the scapular fractures combined with injuries of the ipsilateral SSSC were associated with concomitant injuries with 63 polytraumatized patients (59%) exceeding an Injury Severity Score (ISS) of 16. A summary of clinical data is shown in Table 1.

**Figure 1:**
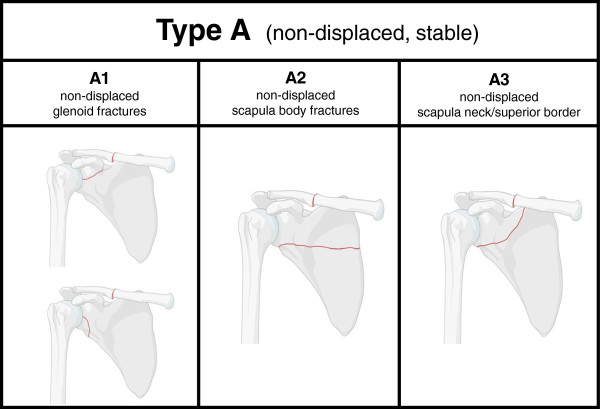
**Classification of non-displaced fractures of the scapula with a stable shoulder girdle (Type A injuries).** Non-displaced fractures of the glenoid are classified as A1 injuries with two subtypes (A1.1 and A1.2), non-displaced fractures of the scapular body are categorized as A2 injuries, fractures involving the superior border as A3 injuries.

Three-dimensional CT-scans of all 107 patients were examined and classified in three groups for the criteria of displacement and instability. Group A consisted of 57 patients (53.3%) with non-displaced fractures judged stable according to the radiographic results. These Type A fractures were further analyzed for typical fracture patterns. Non-displaced fractures of the glenoid were assigned to group A1 and represented 6/57 (10.5%) of all non-displaced, stable injuries. Fractures of the scapular body without involvement of the scapular spine, the scapular neck or the glenoid were classified A2 and represented for 31/57 (54.4%) of all Type A fractures. Non-displaced fractures involving the superior border, scapular spine or neck were defined as A3 fractures if the injury was considered to be stable and represented for 20/57 (35.1%) of all Type A injuries. The classification of non-displaced, stable Type A complex scapular injuries is summarized in Figure 1.

**Figure 2:**
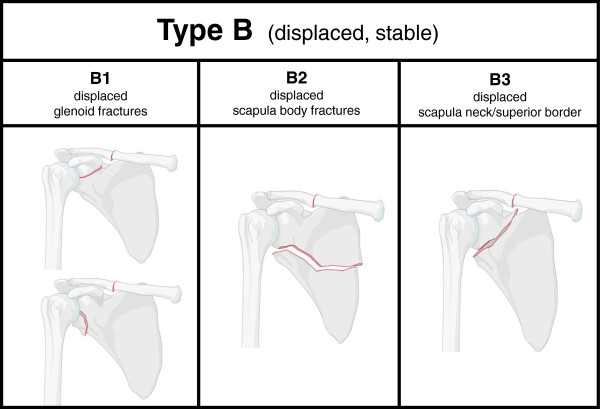
**Classification of displaced fractures of the scapula with a stable shoulder girdle (Type B injuries).** Displaced fractures of the glenoid are classified B1 injuries with two subtypes (B1.1 and B1.2), displaced fractures of the scapular body are categorized as B2 injuries, fractures involving the superior border as B3 injuries. Radiologic definitions of a relevant fracture displacement are given in the text.

Displaced fractures of the scapula with a stable shoulder girdle thus not representing a floating shoulder were categorized as Type B injuries. Injuries of twenty patients of our study group (18.7%) were classified as Type B injuries. Corresponding to non-displaced Type A fractures, displaced fractures of the glenoid were named B1 (65%), displaced fractures of the scapular body B2 (10%) and displaced fractures involving the superior border or the neck of the scapula which were considered to be stable (25%) were defined as B3 injuries. Fracture and displacement patterns of all Type B injuries are summarized in Figure 2.

**Figure 3:**
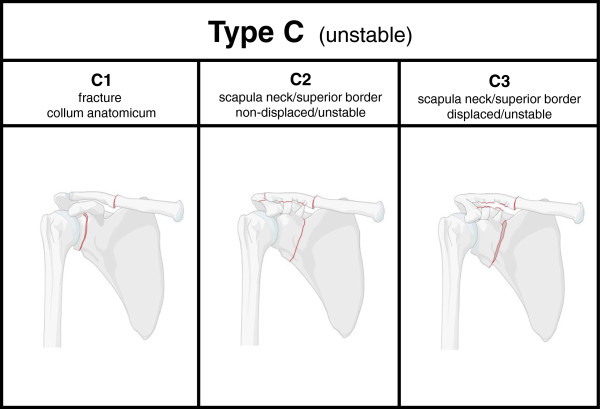
**Classification of fractures of the scapula with an unstable shoulder girdle (Type C injuries).** Fractures of the collum anatomicum are classified as C1 injuries, non-displaced fractures of the superior border with an unstable shoulder girdle are categorized as C2 injuries, displaced fractures involving the superior border of the scapula as C3 injuries.

All unstable injuries or true floating shoulders were classified as Type C injuries. Fractures of the collum anatomicum are considered unstable and were defined as C1 fractures representing 13 out of 30 patients (43.3%) with unstable complex scapula fractures. Three patients (10%) were classified as non-displaced but unstable fractures of the scapular neck combined with at least one more lesion of the SSSC (C2 injuries), fourteen complex scapular injuries (46.7%) were classified as displaced, unstable shoulder girdles and categorized as C3 injuries. The systematic classification is shown in Figure 3.

Injuries of all 107 patients were classified and the treatment was retrospectively evaluated for non-operative or surgical treatment. As expected, almost all patients with Type A injuries were either treated non-surgical (38.6%) or by surgical treatment of the clavicula fracture alone (57.9%). Only two patients received an open reduction and internal fixation of the intra-articular glenoid fracture even though retrospectively the displacement did not meet the criteria for surgery defined for this study. In contrast, seventy per cent of the patients with displaced Type B injuries were treated with open reduction and internal fixation of the scapula (45%) or both, scapula and clavicula (25%). The majority of patients with unstable Type C injuries received an open reduction an internal fixation of the scapula (57%) while in 20 per cent only the clavicle was operated. Although the injuries were retrospectively judged unstable, 23% of the patients were treated non-surgically. This might be due to a high mortality (10%) and a higher rate of brachial plexus lesions (20%) in patients with Type C fractures.

## Conclusions

Since the first description as a fracture of the scapular neck and the ipsilateral clavicula the definition of a “floating shoulder” has been modified several times. Many studies have focused on a better understanding and an improved surgical or non-operative treatment of these complex injuries of the shoulder girdle. However, little agreement and comparative evidence has been achieved and controversies still exist about valid criteria regarding surgical indication and therapy.

High success rates have been reported on non-operative treatment of scapula fractures with concomitant fractures of the ipsilateral clavicula [24]. Edwards and colleagues recommended conservative treatment of complex shoulder injuries within a defined range of displacement of scapula and clavicula [16]. In contrast, Herscovici and co-workers reported of seven patients with fractures of the scapular neck and ipsilateral clavicle treated with open reduction and internal fixation (ORIF) of the clavicle alone and achieved excellent functional results [9]. Recently, Izadpanah and colleagues described good results for an intramedullary stabilization of the clavicle in cases with minor displacement of the scapular neck fracture [25]. Surgical treatment of both scapula and clavicula, is favored by several authors and excellent functional results are reported in various studies [17, 18, 26]. Additionally, several studies investigated the outcome of operative versus non-operative intervention with comparable functional results [11, 19, 20]. However, no study is able to provide measurable criteria for surgical indications. In our opinion, one explanation is the non-comparable description and classification of these complex shoulder injuries. Different injury patterns are often summarized under the definition of a “floating shoulder”.

There is agreement, that non-displaced and stable complex shoulder injuries should be treated conservatively. We classified non-displaced fractures of the scapula with a stable shoulder girdle as Type A injuries. More than half of all injuries were defined as non-displaced and stable (53.3%) and the recommendation of a non-surgical therapy of the scapula in Type A injuries is in accordance to literature [3].

Nearly twenty per cent of our study group presented with displaced fractures of the scapula and a stable shoulder girdle and were classified as Type B injuries. Whereas the displacement of more than 2 mm is considered as an explicit indication for surgical intervention of intra-articular fractures [3, 27, 28], the degree of displacement and angulation as an indication for surgery is controversial for extra-articular fractures. For fractures of the scapular neck or fractures involving the superior border and the spine of the scapula, we consider a displacement of more than 10 mm and an angulation of more than 15–20 degrees an indication for surgery.

We classified true “floating shoulders”, synonymously called fractures of the scapula with an unstable shoulder girdle or unstable double lesions of the SSSC as Type C injuries. Fractures of the collum anatomicum (Type C1) are always considered unstable and are easily detected in 3D-computed tomography. In contrast, in the majority of cases, it is difficult to judge the stability of an injury since not every double lesion of the SSSC is inherently unstable [12, 29]. A diagnostic challenge is the non-displaced but unstable shoulder injury. Classified as Type C2 injury, they account for only ten per cent of all unstable injuries of the shoulder girdle. Although some injuries can primarily be classified as non-displaced but unstable based on undoubtful radiological signs of instability, other Type C2 injuries will only be detected as a secondary displacement of the glenoid after conservative treatment an initially non-displaced Type A3 fracture. In our study group, unstable fractures were considered stable in two cases and conservative treatment failed due to a secondary displacement.

So far, 3-D computed tomography represents the gold standard in the diagnosis of an unstable shoulder girdle. The role of MRI-imaging has not been studied yet and it is possible that additional criteria of instability might be acquired especially in non-displaced injuries. In contrast to these Type C2 injuries, the diagnosis of dislocated, unstable injuries (Type C3) appears to be simple and the indication to a surgical treatment is obvious. In our opinion, all unstable injuries should be treated by open reduction and internal fixation of the scapula via a posterior approach. Although some studies report good results if the clavicle alone is fixed [9, 11, 18, 30], we do not recommend this management. We recommend a direct surgical approach of the scapula for Type B and Type C injuries, the fixation of the clavicle depends on the degree of the clavicula displacement and should be treated independently.

In summary, the described classification is a proposal based on a retrospective study of common injury types often summarized under the term “floating shoulder”. The authors are aware, that no treatment algorithms or recommendations of operative or conservative treatment can be concluded from this data. However, for future studies including short and long time functional outcome it will be necessary to compare similar injury patterns to receive promising results.
